# Brain Network Dynamics Adhere to a Power Law

**DOI:** 10.3389/fnins.2017.00072

**Published:** 2017-02-14

**Authors:** Dardo G. Tomasi, Ehsan Shokri-Kojori, Nora D. Volkow

**Affiliations:** ^1^National Institute on Alcohol Abuse and AlcoholismBethesda, MD, USA; ^2^National Institute on Drug AbuseBethesda, MD, USA

**Keywords:** FCDM, ALFF, lFCD, functional connectivity (FC), graph theory analysis, brain networks, Taylor's law, numerical simulations

## Abstract

The temporal dynamics of complex networks such as the Internet are characterized by a power scaling between the temporal mean and dispersion of signals at each network node. Here we tested the hypothesis that the temporal dynamics of the brain networks are characterized by a similar power law. This realization could be useful to assess the effects of randomness and external modulators on the brain network dynamics. Simulated data using a well-stablished random diffusion model allowed us to predict that the temporal dispersion of the amplitude of low frequency fluctuations (ALFF) and that of the local functional connectivity density (*l*FCD) scale with their temporal means. We tested this hypothesis in open-access resting-state functional magnetic resonance imaging datasets from 66 healthy subjects. A robust power law emerged from the temporal dynamics of ALFF and *l*FCD metrics, which was insensitive to the methods used for the computation of the metrics. The scaling exponents (ALFF: 0.8 ± 0.1; *l*FCD: 1.1 ± 0.1; mean ± SD) decreased with age and varied significantly across brain regions; multimodal cortical areas exhibited lower scaling exponents, consistent with a stronger influence of external inputs, than limbic and subcortical regions, which exhibited higher scaling exponents, consistent with a stronger influence of internal randomness. Findings are consistent with the notion that external inputs govern neuronal communication in the brain and that their relative influence differs between brain regions. Further studies will assess the potential of this metric as biomarker to characterize neuropathology.

## Introduction

During resting-state functional magnetic resonance imaging (rfMRI) (Biswal et al., 1995) the human brain sequentially engages in a series of diverse free-streaming subject-driven mental states supported by different brain networks (Mason et al., 2007; Doucet et al., 2012; Shirer et al., 2012; Liu and Duyn, 2013; Yang et al., 2014). These complex and time-varying functional operations require a dynamic brain network topology to support the context-dependent coordination of neuronal populations (Allen et al., 2014; Zalesky et al., 2014) and its characterization and measurement could facilitate development of clinical biomarkers in neurology and psychiatry (Hutchison et al., 2013). Thus, the temporal dynamics of the human brain connectome (Chang and Glover, 2010; Sakoğlu et al., 2010) provides a new metric of brain function to assess healthy and disease conditions (Calhoun et al., 2014). However, our lack of understanding of the principles governing network dynamics may preclude the interpretation of the observed dynamics, which increases the within-subjects variability of the functional connectivity metrics (Tomasi et al., 2016a,b). A better understanding of how the collective behavior of neuronal communities contributes to the observable dynamics is crucial for the interpretation of the dynamics of functional connectivity.

Previous studies have shown that temporal mean 〈*S*_*i*_〉, and dispersion, σ_*i*_, of the activity at a given node are related through a power law across network nodes (Argollo de Menezes and Barabasi, 2004)

(1) $$\sigma_{i}=a{\langle S_{i}\rangle }^{b},$$

where the scaling exponent, *b*, is a property of the network. Based on theoretical grounds and independent from the topology of the network, *b* equals either ½ or 1, which reflect a competition between the system's internal collective dynamics and temporal changes in the external environment (Argollo de Menezes and Barabasi, 2004). Specifically, in the absence of external modulation, *b* = ½, but when external driving forces become dominant, *b* = 1. For instance, whereas the network of internet routers is characterized by *b* = ½, the network of highways and the World Wide Web are characterized by *b* = 1. However, empirical evidence from ecology, where (1) describes the spatiotemporal variability of natural populations, supports the existence of intermediate *b*-values (Taylor, 1961) suggesting that meaningful temporal dynamic require ½ < *b* < 1.

Inasmuch as brain networks have scale-free (Barabasi and Albert, 1999; Eguíluz et al., 2005) and small-world (Watts and Strogatz, 1998) properties exhibited by complex networks we hypothesized that the mean and σ of FC properties such as ALFF, the amplitude of the low frequency fluctuations (Yang et al., 2007) or *l*FCD, the local degree of connectivity (Tomasi and Volkow, 2010) would reveal the characteristic power scaling properties exhibited by other complex networks. Specifically, we hypothesized that the mean and σ would be related by the power law (1) and that different brain networks would exhibit different scaling exponents reflecting differential balance between internal randomness (random firing) and external inputs (non-random firing). We selected functional connectivity (ALFF and *l*FCD) metrics rather than raw signals because the mean and dispersion values of the BOLD-fMRI signals are not expected to be in agreement with Equation (1).

## Methods

To interpret the observed power scaling law (1), we study a simple dynamical model based on random diffusion. Using this model and functional connectivity information extracted from rfMRI datasets, we assessed the validity of Equation (1) in the context of brain functional connectivity. However, since direct application of Equation (1) to the mean and dispersion values of the raw fMRI time series is meaningless (the MRI signal mainly reflects tissue properties such as water density and T1 and T2 relaxation rates, which do not change as a function of time; the BOLD signal is zero-mean by definition), we simulated the temporal dynamics of ALFF and *l*FCD.

### Model

Similar to previous studies (Argollo de Menezes and Barabasi, 2004), to model the signal *S*(*t*) we simulated the random diffusion of W walkers (messages) on a network of N nodes described by its adjacency matrix, *A*_*ij*_. Each walker was placed at a randomly chosen network node from which it departed randomly along one of the edges of that node in the next time step. This diffusion process was independently repeated 1,200 times and we recorded the number of incoming visits by various walkers at each network node to compute the time-varying signal at each node, *S*_*i*_(*t*). Temporal fluctuations in W were used to simulate externally induced modulations in *S*_*i*_(*t*), which for random networks and scale-free networks results in *b* = 1 exponent in (1) (Argollo de Menezes and Barabasi, 2004). Thus we varied the number of walkers as a function of time as: W(*t*) = W + ξ(*t*), where ξ(*t*) was a uniformly distributed random variable in the interval [−ΔW, ΔW], with ΔW = k^*^10^3^ and *k* = 0,1,2,…, 9, and W = 10^4^.

### Simulations

**Figure 1:**
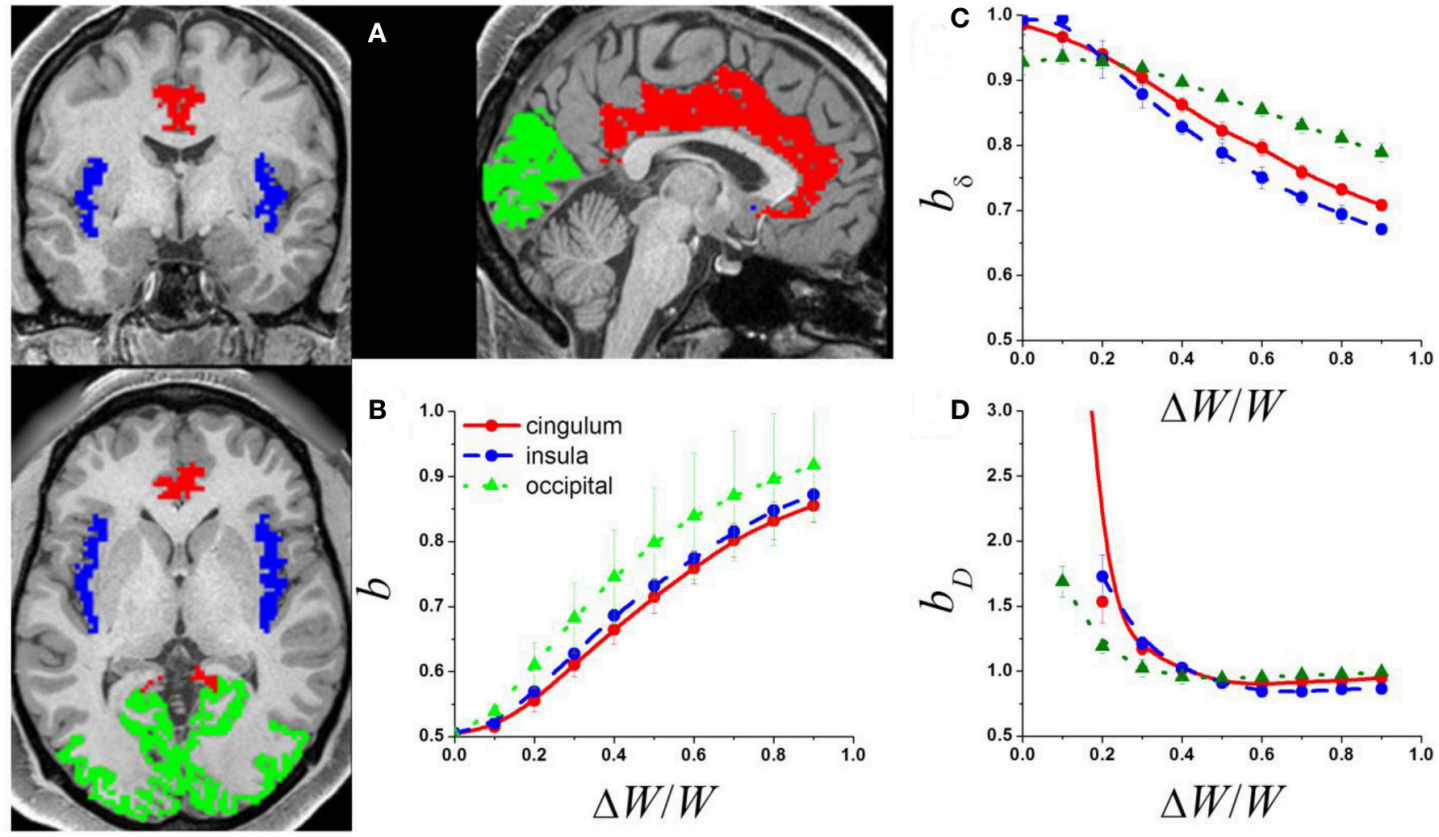
**(A)** Exemplary single-subject structural data showing occipital (green) cingulate (red) and insular (blue) gray matter networks used to compute the adjacency matrix from the corresponding rfMRI datasets. The adjacency matrices of these networks and a random diffusion model were used to produce simulated signal fluctuations, *S*_*i*_, with variable relative external modulation (ΔW/W) at each network node. The scaling exponent, *b*, was obtained by linear fitting the temporal mean and dispersion values of 〈*S*_*i*_〉 in a log-log plot. **(B)** Average *b* across network nodes and 7 subjects as a function of ΔW/W for the 3 different networks. Scaling exponent as a function of ΔW/W for the 3 different networks for: **(C)** the amplitude of the signal fluctuations, *b*_δ_; and **(D)** the degree of the functional connectivity, *b*_D_ (see Methods).

The FreeSurfer gray matter parcellations (wmparc.2.nii) for 7 randomly selected MRI datasets were used to determine imaging voxels in the occipital, cingulate and insular networks (Figure 1A). The occipital network comprised bilateral cuneus, lateral occipital, lingual, and pericalcarine cortices (number of nodes/voxels, *N* = 8,200 ± 600). The cingulate network comprised bilateral rostral anterior, caudal anterior, isthmus, and posterior cingulate (*N* = 3,100 ± 400). The insular network comprised the bilateral insula (*N* = 2,300 ± 100). The Pearson correlation was used to compute correlation matrices reflecting the functional connectivity between voxels within each network for each subject. A correlation threshold *R* = 0.2 (*p* < 0.05) was used to compute the corresponding binary adjacency matrices. We implemented the diffusion model described above (Argollo de Menezes and Barabasi, 2004). We assumed the signal is proportional to the rate of incoming messages at each node as a function of time, which was simulated using 1,200 steps.

To simulate the dynamics in the amplitude of the signal fluctuations, δ_*i*_, at each node we segmented the *S*_*i*_(*t*) data (1,200 time points) into 23 epochs (window length: 100 time points; window shift: 50 time points) using a popular rectangular sliding window approach (Chang and Glover, 2010). The temporal standard deviation of *S*_*i*_(*t*) during each epoch was used to estimate δ_*i*_. Degree, D_*i*_, the number of links connected to a network node (Rubinov and Sporns, 2010), was computed for each of the 23 epochs of the synthetic *S*_*i*_(*t*) data using a correlation threshold, *R* > 0.5 (*p* < 10^−7^). The linear model log(σ_*X*_) = log(*a*) + *b*log〈*X*〉 with 2 freely adjustable parameters: log(*a*) and *b*, was used to fit the power law (1) to the temporal mean and dispersion values of the dynamic δ and *D* metrics (*X*).

### Datasets

To test the predictions of the random diffusion model we analyzed rfMRI datasets drawn from the Human Connectome Project (HCP; http://www.humanconnectome.org/). No experimental activity with any involvement of human subjects took place at the author's institutions. The 66 participants (age: 30 ± 3 years; 32 females; Subject IDs: 100408, 103515, 103818, 105115, 105216, 106319, 110411, 118730, 118932, 119833, 120212, 122317, 123117, 125525, 127933, 128632, 129028, 130013, 131924, 133625, 133827, 133928, 134324, 136833, 137128, 138231, 138534, 140824, 142828, 143325, 144226, 149337, 149539, 150423, 151526, 153429, 156637, 158540, 159239, 159340, 160123, 161731, 162329, 163129, 165840, 167743, 172332, 178950, 182739, 191437, 192439, 192540, 194140, 197550, 199150, 199251, 200614, 201111, 210617, 217429, 249947, 250427, 255639, 304020, 307127, 329440) of the WU-Minn HCP Q1 data release included in this study provided written informed consent according to procedures approved by the IRB at Washington University in St. Louis.

Resting-state (eyes open) functional images were acquired using a gradient-echo-planar (EPI) sequence with multiband factor 8, TR 720 ms, TE 33.1 ms, flip angle 52°, 104 × 90 matrix size, 72 slices, 2 mm isotropic voxels, and 1200 timepoints (Smith et al., 2013; Uğurbil et al., 2013). Scans were repeated twice using different phase encoding directions (LR and RL) on each of two imaging sessions (REST1 and REST2) collected on different days. The “minimal preprocessing” datasets, which include gradient distortion correction, rigid-body realignment, field-map processing, spatial normalization to the stereotactic space of the Montreal Neurological Institute (MNI), high pass filtering (1/2,000 Hz frequency cutoff) (Glasser et al., 2013), independent component analysis-based denoising (Salimi-Khorshidi et al., 2014), and brain masking were used in this study.

### Preprocessing

Framewise displacements, FD, computed for every time point from head translations and rotations using a radius of *r* = 50 mm (Power et al., 2012) did not differ between MRI sessions or phase encoding directions across subjects (*p* > 0.2, paired *t*-test; 〈FD〉 = 0.176 ± 0.05 mm). Scrubbing was not implemented to preserve the frequency spectra used for the computation of ALFF. Multilinear regression of head translations and rotations were used to minimize motion related fluctuations in the MRI signals (Tomasi and Volkow, 2010). Standard 0.01–0.08 Hz band-pass filtering was used to minimize physiologic noise of high frequency components.

### Dynamic ALFF and *l*FCD

The average of the power spectrum's square root in the 0.01–0.08 Hz low frequency bandwidth was used to compute the ALFF (Yang et al., 2007). Functional connectivity density mapping was used to compute the *l*FCD (Tomasi and Volkow, 2010) at three different thresholds *R* > 0.3, 0.4 and 0.5. A sliding window approach (Chang and Glover, 2010) with two different window lengths (72s and 144s) and two different window shapes (rectangular and Hamming) was used to compute dynamic ALFF and *l*FCD maps with 2-mm isotropic resolution at two different temporal resolutions (36s and 72s). The window shift was set as half of the window length.

### Region-of-interest (ROI) analysis

**Figure 2:**
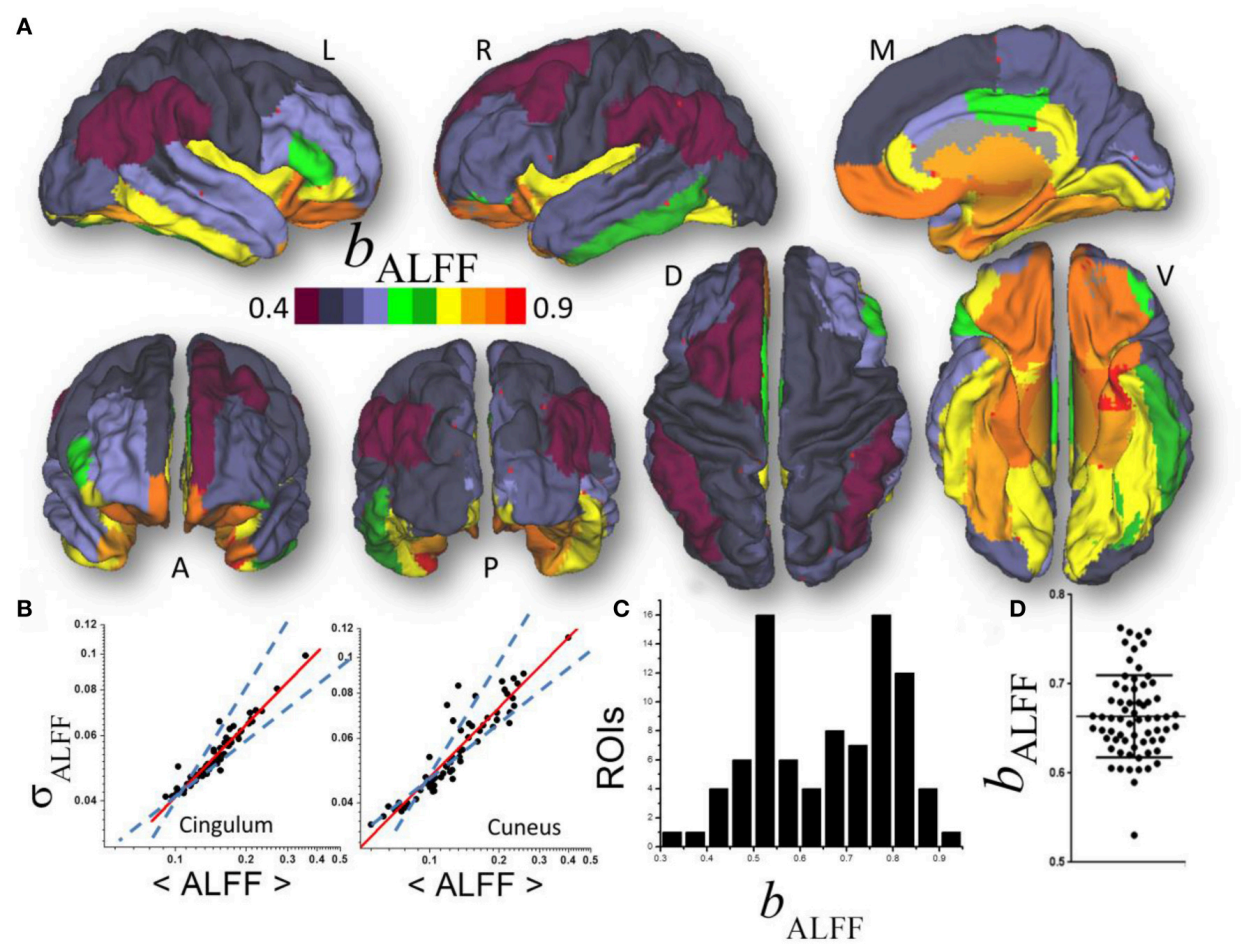
**(A)** Average scaling exponent (*b*_ALFF_) for the temporal dynamics of the amplitude of low frequency fluctuations (ALFF) computed across nodes independently for each of the individual anatomical ROIs, superimposed on left (L), right (R), dorsal (D), medial (M), ventral (V) anterior (A), and posterior (P) views of the cerebral surface of the PALS_B12 template. **(B)** Scatter plots showing the good agreement across 66 subjects (dots) between the power law (1) (red line) and the dynamics of ALFF which is characterized by its temporal mean, 〈ALFF〉, and dispersion, σ_ALFF_. Dashed lines are the upper (*b*_δ_ = 1; pure randomness) and lower (*b*_δ_ = ½; pure external modulation) limits for the scaling exponent. **(C)** Frequency count histogram reflecting the probability distribution of *b*_ALFF_ across cortical and subcortical gray matter ROIs. **(D**) Scatter plot demonstrating the variability of *b*_ALFF_ across 66 young adults.

**Figure 3:**
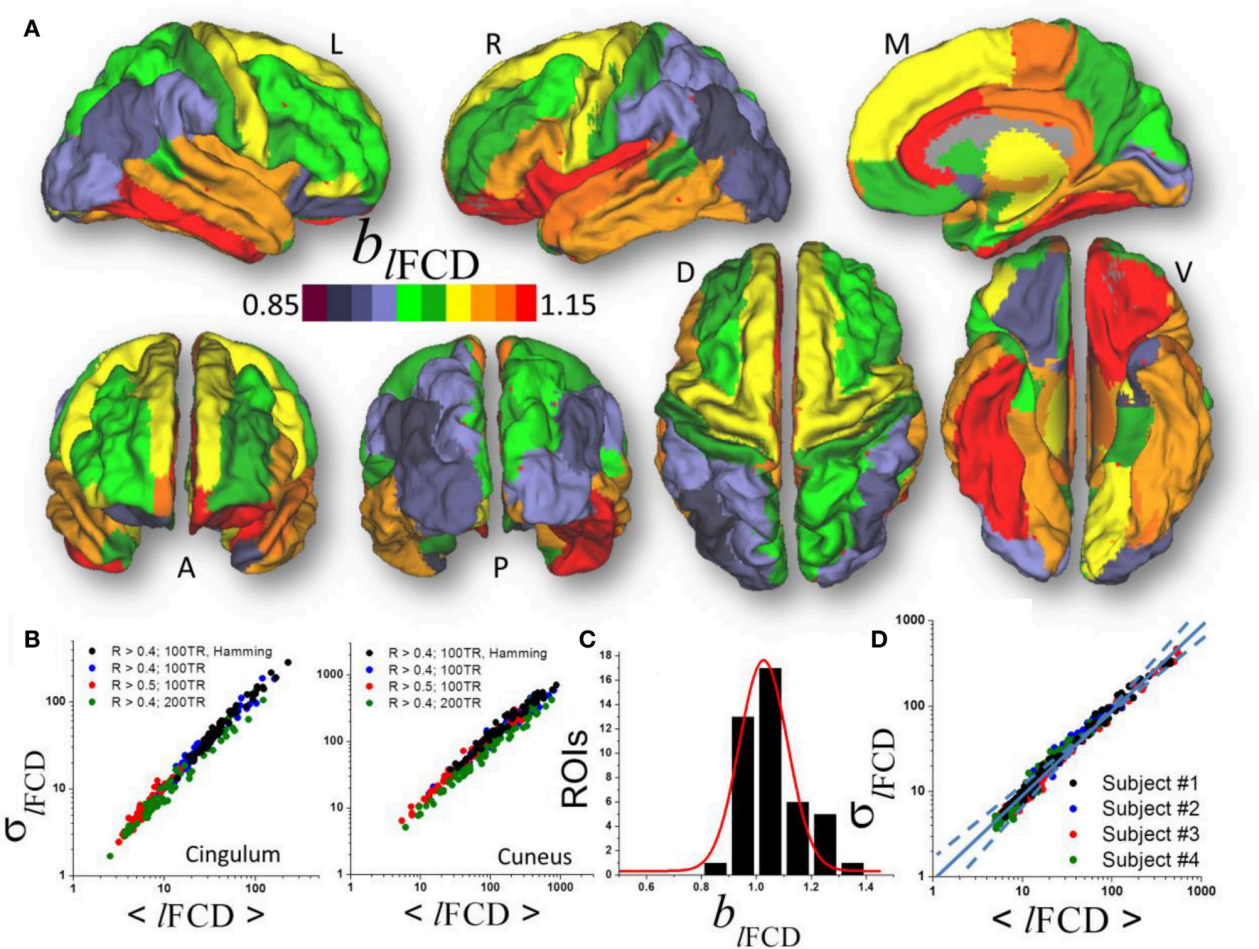
**(A)** Average scaling exponent (*b*_*l*__FCD_) for the temporal dynamics of the local functional connectivity density (*l*FCD) computed across nodes independently for each of the individual anatomical ROIs superimposed on left (L), right (R), dorsal (D), medial (M), ventral (V) anterior (A), and posterior (P) views of the cerebral surface of the PALS_B12 template. **(B)** Scatter plots across 66 subjects showing the robustness of the power law (1) that reflects the dynamics of *l*FCD to changes in correlation thresholds, sliding window lengths and shapes. **(C)** Frequency count histogram reflecting the probability distribution of *b*_*l*__FCD_ across cortical and subcortical gray matter ROIs. **(D)** Scatter plot demonstrating the moderate differences power law (computed across 86 ROIs) in four typical subjects.

To test the power law (1) we contrasted scaling factors for the simulated signal fluctuations (δ) and degree (D) against those for ALFF and *l*FCD. Since *l*FCD has high sensitivity and specificity for gray matter (Tomasi et al., 2016c), the FC metrics were averaged within the anatomical gray matter regions-of-interest for each individual to minimize confounds arising from the variability of the folding patterns of cortical gray matter. Specifically, the FreeSurfer gray matter parcellations (wmparc.2.nii) were used as ROIs to compute the averages of the temporal mean and dispersion values of ALFF(*t*) and *l*FCD(*t*) within 34 cortical and 9 subcortical gray matter regions in each brain hemisphere. A probabilistic atlas for each of the gray matter parcellations was developed by averaging each of the gray matter parcellations across subjects independently, and used to display ROI results (i.e., *b*_ALFF_ or *b*_*l*__FCD_) in the MNI stereotactic space (Figures 2A, 3A).

### Statistical methods

The linear model log(σ) = log(*a*)+*b*log〈*X*〉 with 2 free adjustable parameters: log(*a*) and *b*, was used to fit the power law (1) to the temporal mean and dispersion values of the dynamic ALFF and *l*FCD metrics (*X*). Paired *t*-test was used to assess within subjects differences in *b*_ALFF_ and *b*_*l*__FCD_ as a function of session, phase encoding direction, correlation threshold, and window length and shape. Two samples *t*-test and Pearson correlation were used to assess gender and aging effects on *b*_ALFF_ and *b*_*l*__FCD_.

## Results

### Simulations

The power law (1) fitted well (*R*^2^ > 0.8) the temporal mean and standard deviation values of *S*_*i*_(*t*) across nodes. The *b* exponents increased monotonically with ΔW, which is consistent with the notion that internal randomness (diffusion) and external modulation (ΔW) proportionally alter *S*_*i*_(*t*) in the network (Argollo de Menezes and Barabasi, 2004). Thus, ΔW contributed to the temporal variability of the signal at each network node, gradually increasing *b* from ½ to 1 in all three brain networks (Figure 1B) as it occurs in other complex networks. Thus, if its magnitude is significant (ΔW ~ ½ 〈W〉), the external modulation can dominate the dynamics of *S*_*i*_(*t*).

The mean and dispersion values of δ_*i*_ computed across epochs were also in good agreement with the power law (1). Our simulations suggest that, *b*_δ_ ~ 1, when internal randomness dominates over the external modulations (Figure 1C). However, *b*_δ_ decreased with the amplitude of the external modulation and was constrained in the interval [0.5, 1]. Similarly, the mean and dispersion values of D_*i*_ computed across epochs were in good agreement with the power law (1). Our simulations suggest that *b*_D_ ~ 1 when the external modulation dominates over the internal randomness, but *b*_D_ increases significantly above 1 when the relative weight of the external modulation decreases (Figure 1D). The power law failed to fit the data when internal randomness dominated over the external modulation (ΔW/W > ½) suggesting lack of association between the mean and dispersion values of D in this regime.

### Amplitude of fluctuations

A linear fit of whole-brain average and dispersion values of ALFF on a log-log plot computed across nodes demonstrated good agreement between Equation (1) and the dynamic amplitude of the signal fluctuations in each of the individual ROI (*b*_ALFF_ = 0.66 ± 0.16, mean ± standard deviation; 28 < *t*-score <294; *P* < 1E-37; Figure 2A). Consistent findings emerged from average and dispersion values within anatomical regions, independently for each of the 86 gray matter ROIs (*R*^2^ > 0.8; Figures 2B,C). The average scaling exponent was not different for subcortical (cerebellum, thalamus, caudate, putamen, pallidum, hippocampus, amygdala, accumbens and ventral diencephalon; *b*_ALFF_ = 0.78 ± 0.04, mean ± standard error) than for cortical (*b*_ALFF_ = 0.81 ± 0.07) regions (*p* > 0.4), independent of session, phase encoding direction (LR vs. RL), sliding window length (72s vs. 144s) and shape (rectangular vs. Hamming).

There were no significant differences in *b*_ALFF_ across subcortical regions. However, *b*_ALFF_ varied significantly across cortical regions. Specifically, the scaling exponent was higher for limbic (cingulum, orbitofrontal, parahippocampal and entorhinal) and visual (lingual, fusiform and pericalcarine) areas, the temporal and insular cortices and pars orbitalis (*b*_ALFF_ = 0.89 ± 0.02) than for occipital (cuneus, lateral occipital), parietal (inferior, superior, precuneus, postcentral), language (opercularis, triangularis, supramarginal) and prefrontal (paracentral, precentral, rostral, middle and superior frontal) areas (*b*_ALFF_ = 0.72 ± 0.03; *p* < 10^−9^; Figure 2B). The scaling exponent had normal distribution (center *b*_ALFF_ = 0.80; width = 0.16) across the 86 gray matter ROIs (*R*^2^ = 0.999, Gaussian fit; Figure 2C).

Significant between-subjects variability in the scaling exponent emerged from the data when we fitted Equation (1) to the mean and dispersion values of ALFF across the 43 ROIs, independently for each individual (*b*_ALFF_ = 0.66 ± 0.05; Figure 2D) and with similar robustness (*R*^2^ > 0.96). The scaling exponent slightly decreased with age (slope = −0.03/decade; *R* = −0.234; *p* = 0.03, one-tailed). However, there were no significant gender differences (*p* > 0.77; two-tailed two-sample *t*-test) in *b*_ALFF_.

### Local degree

Similar to ALFF, a linear fit of whole-brain average and dispersion values of *l*FCD on a log-log plot computed across nodes demonstrated good agreement between Equation (1) and the local degree of brain functional connectivity in each of the individual ROI (*b*_*l*__FCD_ = 1.05 ± 0.17, mean ± standard deviation; 43 <t-score <179; *P* <3E-49; Figure 3A). Average and dispersion values within anatomical regions showed consistent findings with those from the whole-brain analysis (*R*^2^ > 0.8; Figure 3B). The average scaling exponent was higher for subcortical (*b*_*l*__FCD_ = 1.23 ± 0.09, mean ± standard error) than for cortical (*b*_*l*__FCD_ = 1.06 ± 0.10) regions (*p* < 10^−3^, two-tailed two-sample *t*-test), independent of the correlation threshold used in the computation of the *l*FCD (*R* > 0.3, 0.4, or 0.5), session, phase encoding direction, window length (72s vs. 144s) and shape.

For *l*FCD, the scaling exponent was higher for limbic (cingulum, orbitofrontal, parahippocampal, and entorhinal), language (opercularis, orbitalis, triangularis), temporal (inferior, middle superior), and frontal (paracentral, superior and pole), insula and fusiform gyrus (*b*_*l*__FCD_ = 1.13 ± 0.07) than for occipital (cuneus, lateral occipital, lingual and pericalcarine), parietal (inferior, superior, precuneus, supramarginal, paracentral, postcentral), prefrontal (precentral, rostral, middle, and superior) and temporal (entorhinal temporal pole, transverse) areas (*b*_*l*__FCD_ = 0.98 ± 0.06; *p* < 10^−6^; Figure 3B). Across the 86 gray matter ROIs the scaling exponent had a right-skewed distribution with peak at *b*_*l*__FCD_ = 1.03 and width = 0.17 (*R*^2^ = 0.95, Gaussian fit; Figure 3C). Fitting mean and dispersion values of *l*FCD across the 43 gray matter ROIs, independently for each subject, revealed modest between-subjects variability in the scaling exponent (*b*_*l*__FCD_ = 1.09 ± 0.06; Figure 3D), and *b*_*l*__FCD_ did not show significant age or gender differences (*p* > 0.23).

**Figure 4:**
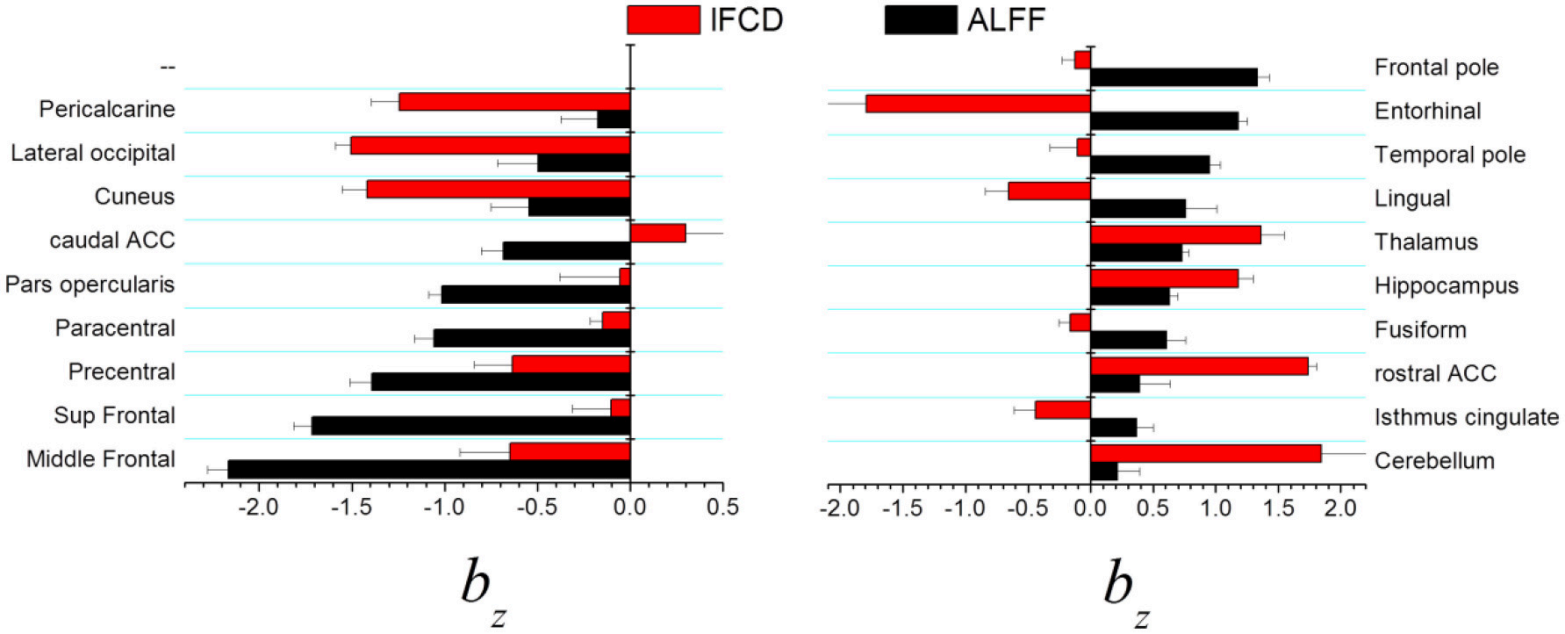
**Brain regions showing statistically significant differences in standardized scaling exponents, *b*_*z*_, for ALFF and *l*FCD at *p* < 0.001**. ACC, anterior cingulate cortex.

In visual areas (pericalcarine, lateral occipital, and cuneus) the standardized scaling factors *b*_z_ were lower than average and were significantly lower for *l*FCD than for ALFF (*p* < 0.0005, *t*-test; Figure 4, left). In prefrontal regions (middle Frontal, superior frontal, precentral, paracentral, pars opercularis, and caudal anterior cingulate), the lower than average *b*_*z*_ was lower for ALFF than for *l*FCD (*p* < 0.001). In frontal and temporal poles, entorhinal and lingual cortex showed the higher than average *b*_z_ was higher for ALFF than for *l*FCD (*p* < 0.0001; Figure 4 right). In anterior (rostral) and posterior (isthmus) cingulate, fusiform gyrus and subcortical regions (hippocampus, thalamus and cerebellum) the higher than average *b*_z_ was higher for *l*FCD than for ALFF (*p* < 0.0006).

### Effect of bandpass filtering

**Figure 5:**
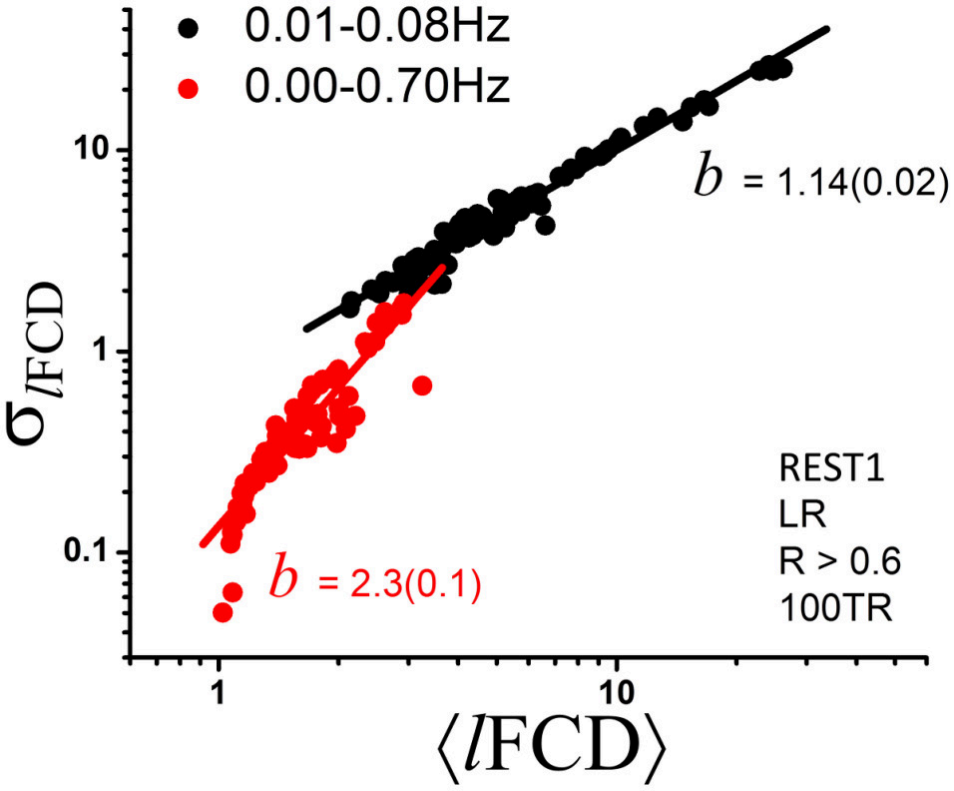
**Scatter plot demonstrating the effect of bandpass filtering on the power scaling (Equation 1) of *l*FCD across ROIs**. The fitted slope, b, the scaling factor in Equation (1), is significantly steeper without bandpass filtering (standard errors in parenthesis) suggesting increased level of randomness (see Figure 1D).

Given that frequency information may be of interest and that the ICA-FIX denoising procedure can remove a significant fraction of the physiological noise of respiratory origin (Salimi-Khorshidi et al., 2014), we also computed dynamic *l*FCD measures without 0.01–0.08 Hz bandpass filtering to assess the effect of higher frequencies on the power scaling law (Equation 1). Without bandpass filtering the scaling exponent *b* of the dynamic *l*FCD metrics was significantly larger than with bandpass filtering (*p* < 0.0001; Figure 5), and the agreement between the data and Equation (1) was significantly reduced [*R*^2^ = 0.82 (without) and 0.96 (with bandpass filtering)].

## Discussion

Here we show for the first time that the mean and the dispersion values of dynamic FC metrics such as ALFF or *l*FCD are linked by a power law (1). This characteristic of complex networks such as rivers and highways networks, the Internet and the World Wide Web (Argollo de Menezes and Barabasi, 2004), and many biological systems (Taylor, 1961), reflects the competition between the system's internal collective dynamics and changes in the external environment. This strongly suggests that the dynamics of the FC metrics embeds important functional information, a possibility previously highlighted (Hutchison et al., 2013; Calhoun et al., 2014; Rashid et al., 2014; Hutchison and Morton, 2015), which could help in the development of biomarkers of brain function.

Our simulations were based on a random diffusion model previously proposed by Argollo de Menezes and Barabasi to explain the power scaling between the mean and the dispersion of the signals observed in natural and technological networks (Argollo de Menezes and Barabasi, 2004). Whereas the approach by Argollo de Menezes and Barabasi was based on random and scale-free networks (Barabasi and Albert, 1999; Barabási, 2009), the present approach was based on real FC networks directly extracted from *in vivo* resting fMRI data. The scaling exponents for the brain in the present work are consistent with those obtained previously in random and scale-free networks (Argollo de Menezes and Barabasi, 2004).

Here we extended the random diffusion model in order simulate the amplitude of spontaneous signal fluctuation and the degree of connectivity. Our simulations suggest that under pure randomness (i.e., without external driving forces, ΔW = 0) the mean and the dispersion values of the amplitude of signal fluctuations and degree are associated by power laws with scaling exponents *b*_δ_ = 1 and *b*_D_ > 1, respectively. However, under the influence of dynamic external modulations (ΔW/W ~ 1), *b*_δ_ < 1 and *b*_D_ = 1 characterize the dynamic behavior of the signal fluctuations and degree. The analysis of variability of resting-state fMRI datasets from the HCP database shows a range of scaling exponents for ALFF (0.5 < *b*_ALFF_ < 1) and for *l*FCD (1 < *b*_*l*__FCD_), which is consistent with the presence of dynamic external modulations of brain activity (0.5 < *b*_δ_ < 1) and the corresponding degree (1 < *b*_D_). Overall, our findings are also consistent with the existence of dynamic modulations of brain activity that may reflect orchestrated dynamic neural processing (Yu et al., 2012; Allen et al., 2014; Gonzalez-Castillo et al., 2015).

This is the first study to document differences in scaling exponents between brain regions. Multimodal association areas (opercularis, triangularis, rostral, middle and superior frontal, precentral and paracentral, inferior and superior parietal and precuneus), somatosensory (supramarginal, postcentral) and visual (cuneus, lateral occipital) unimodal association areas showed low scaling exponent both for ALFF (*b*_ALFF_ ~ 0.7) and for *l*FCD (*b*_*l*__FCD_ ~ 1). These findings suggest that the dynamics of the FC metrics was driven by external inputs (ΔW/W > ½) rather than by internal random processes (ΔW/W <0.5; Figures 1C,D), which is also consistent with the existence of dynamic modulations of resting brain activity (Yu et al., 2012; Allen et al., 2014; Gonzalez-Castillo et al., 2015). The multimodal cortex is highly interconnected with higher-order association areas involved in cognition and motor planning (Goldman-Rakic, 1988). Thus dynamic engagement of functional connectivity hubs in multimodal and unimodal association cortices may explain the low scaling exponent in these regions. On the other hand, limbic and subcortical regions exhibited relatively higher scaling exponents (*b*_ALFF_ ~ 0.8 and *b*_*l*__FCD_ ~ 1.2) suggesting a stronger influence of internal randomness in the resting dynamics of the FC metrics in these regions.

We identify regional differences in the influence of internal randomness for different FC metrics. The direct comparison of standardized measures suggests a weaker influence of randomness in visual areas for *l*FCD than for ALFF and in prefrontal areas for ALFF than for *l*FCD, and a stronger influence of randomness in subcortical and limbic regions for *l*FCD than for ALFF. ALFF and *l*FCD reflect different network properties. Whereas ALFF is proportional to the BOLD signal fluctuations that reflect neuronal communication (Logothetis et al., 2001), the synchronous fluctuations of local communities measured by *l*FCD reflects the local degree of connectivity (Tomasi and Volkow, 2010).

The scaling exponent for ALFF, and to a lesser extent for *l*FCD, showed significant variability (Δ*b*_ALFF_ = 12%; Δ*b*_*l*__FCD_ = 9%) across subjects suggesting that the dynamics of the *b* has potential as a biomarker for psychiatry and neurology. To illustrate the potential of this metric here we show that even in a relatively small sample (66 subjects) with narrow age range (22–35 years), *b*_ALFF_ is sensitive to aging effects, consistent with previous studies in large samples (~1000 subjects) with wide age range (17–82 years) that documented age-related decreases in FC (Biswal et al., 2010; Tomasi and Volkow, 2012).

The scaling exponent for *l*FCD increased significantly above 1 when frequencies other than those in the 0.01–0.08 Hz band were not removed from the data. At the same time, the agreement with a power scaling was reduced when Equation (1) was fitted to the data without bandpass filtering. This likely reflects the introduction of additional randomness and is consistent with increased noise level and lack of additional information at higher frequencies than those in the 0.01–0.08 Hz band.

The brain normally operates under certain level of randomness that is important for multiple operation including perception and decision-making. The relevance of internal neuronal noise has been most extensively studied for visual perception (Brascamp et al., 2006; Kim et al., 2006). Theoretical studies have also shown that randomness may influence behavioral responses when there are multiple routes to action and suggested that noise generated by random firing rates of neurons can be used to predict a decision (Rolls, 2012). Since limbic and subcortical regions support automatic, implicit decision making (Floresco et al., 2008; Mitchell, 2015) the higher scaling exponents in these regions suggests an important role of randomness in implicit decision making processes. The sensitivity to randomness of *b* could be useful for studying psychiatric disorders such as autism, which is associated with increased randomness of endogenous brain oscillations (Lai et al., 2010).

### Study limitations

Note that *b* = ½ emerges either from diffusion or from flow models, independently of the number of steps in the diffusion model, and from random networks as well as from scale-free networks. This indicates that *b* = ½ is not a particular property of the random diffusion model, but it is shared by several dynamic processes (Argollo de Menezes and Barabasi, 2004). Our computational resources did not allow demanding whole brain network simulations at 2-mm isotropic resolution (~10^5^ nodes/voxels). Thus, our simulations suggesting that when internal randomness dominates over the external modulations (ΔW/W ~ 0) *b*_δ_ ~ 1 and *b*_D_ > 1, but when external modulations dominate over internal randomness (ΔW/W ~ 1) *b*_δ_ ~ 0.5 and *b*_*D*_ ~ 1 are limited to the 3 exemplary networks in this work. However, it is likely that they apply also to the whole brain. Instrumental noise likely resulted in overestimations of intrinsic randomness in subcortical regions for which the 32 channel RF coil used by the HCP has low sensitivity. Since the theoretical model was developed across network nodes, the interpretation of the power law across ROIs and subjects could be considered controversial. Our empirical evidence, however, suggests that the temporal mean and standard deviation values of dynamic functional connectivity metrics also adhere to a power law computed across ROIs or subjects, which are consistent with the power law computed across nodes (i.e., across nodes of each individual ROI, *b*_*l*__FCD_ = 1.05 ± 0.17 mean ± standard deviation; across the 86 gray matter, ROIs *b*_*l*__FCD_ = 1.03 ± 0.17; across 66 subjects, *b*_*l*__FCD_ = 0.98 ± 0.16). This suggests similar effects of randomness and external modulators on power scaling factors computed across network nodes, ROIs or subjects.

Dynamic *l*FCD is restricted to the local functional connectivity cluster. We did not assess the dynamics of global functional connectivity density (*g*FCD) because at high spatiotemporal resolution *g*FCD is extremely demanding and beyond our computational resources. However, this is not a strong limitation because previous studies have shown that the *l*FCD and *g*FCD metrics are proportional to one another (Tomasi and Volkow, 2011).

## Author contributions

DT designed the study, carried the analyses and wrote the manuscript. ES developed imaging tools and wrote the manuscript. NV wrote the manuscript.

### Conflict of interest statement

The authors declare that the research was conducted in the absence of any commercial or financial relationships that could be construed as a potential conflict of interest.
